# Semi-field evaluation of aquatic predators for the control of *Anopheles funestus* in rural south-eastern Tanzania

**DOI:** 10.1186/s12936-024-05055-1

**Published:** 2024-08-02

**Authors:** Herieth H. Mahenge, Letus L. Muyaga, Joel D. Nkya, Andrew D. Kafwenji, Yohana A. Mwalugelo, Najat F. Kahamba, Halfan S. Ngowo, Emmanuel W. Kaindoa

**Affiliations:** 1 Environmental Health and Ecological Sciences Department, Ifakara Health Institute, P. O. Box 53, Ifakara, Tanzania; 2 School of Life Sciences and Bio Engineering, The Nelson Mandela, African Institution of Science and Technology, Tengeru, Arusha, United Republic of Tanzania; 3 School of Biodiversity, One Health & Veterinary Medicine, University of Glasgow, Glasgow, UK; 4 Department of Biomedical Sciences, Jaramogi Oginga Odinga University of Science and Technology, P. O Box 210-40601, Bondo, Kenya; 5 Faculty of Health Sciences, School of Pathology, The Centre for Emerging Zoonotic and Parasitic Diseases, Wits Research Institute for Malaria, National Institute for Communicable Diseases, University of the Witwatersrand, Johannesburg, South Africa

**Keywords:** *Anopheles funestus* group, Predators, Aquatic habitats, Efficacy, Malaria transmission, Biological control and Ifakara Health Institute

## Abstract

**Background:**

Biological control is a promising alternative or complementary approach for controlling vector populations in response to the spread of insecticide resistance in malaria vectors. This study evaluated the efficacy of three selected potential predators on the density and fitness parameters of *Anopheles funestus* larvae in rural Tanzania.

**Methods:**

Common predator families Aeshnidae (dragonflies), Coenagrionidae (damselflies), and Notonectidae (backswimmers) and *An. funestus* group larvae were collected from natural aquatic habitats in rural south-eastern Tanzania. Predators were starved for 12-h while *An. funestus* larvae were given fish food before starting the experiment. *Anopheles funestus* larvae were placed into artificial habitats containing predators, exposing them to potential predation*.* The number of surviving *An*. *funestus* larvae were counted every 24-h. An emergence traps were placed at the top of artificial habitats to capture emerging mosquitoes. Emerged mosquitoes were monitored until they died. Female wings were measured and used as a proxy for body size. Generalized linear mixed models (GLMM) with binomial variates at 95% CI and Cox proportional hazard models were used to assess the proportion of dead mosquitoes and the daily survival determined.

**Results:**

There were significant differences in the number of emerged mosquitoes between the treatment and control groups (P < 0.001). Thus, all predator species played a significant role in reducing the density of *An*. *funestus* mosquitoes (P < 0.001). Furthermore, these predators had notable effects on the fitness parameters and survival of emerged mosquitoes (P < 0.001). Among the three predators studied, Coenagrionidae (damselflies) were most efficient followed by Notonectidae (backswimmers), with Aeshnidae (dragonflies) being the least efficient.

**Conclusion:**

Selected aquatic predators have the potential to reduce the survival and density of *An. funestus* larvae. They might eventually be included within an integrated malaria vector control strategy, ultimately leading to a reduction in malaria transmission.

## Background

Despite significant efforts made to reduce its burden, malaria continues to be one of the major public health challenges in Africa [1]. For example, there were 247 million malaria cases and 619,000 deaths globally, with sub-Saharan Africa contributing to about 95% and 96% of these cases and deaths, respectively [2]. Though the core vector control tools, insecticide-treated nets (ITNs), indoor residual spraying (IRS) and effective diagnosis and treatment have significantly reduced malaria cases and deaths across Africa since 2001 [3], the previous World Malaria report shows that transmission has stalled due to major control limitations associated with COVID-19 disruptions [4], insecticide resistance [5], behaviour change in mosquitoes [6], as well as human behaviour and activities [7, 8]. Due to these challenges, achieving malaria elimination will be difficult unless additional methods are developed that complement existing interventions [9].

Biological control of mosquitoes is recognized as one of the best approaches for controlling malaria vectors in endemic countries [5, 10, 11]. Different aquatic invertebrate predators including, Aeshnidae (dragonflies) [12–14], Notonectidae (backswimmers) [12–14] and Dytiscidae (predaceous diving beetles) [13–16], Coenagrionidae (damselflies) [13, 14], Belostomatidae (giant water bugs) [14], Corixidae (water boatmen) [14], Ranidae (tadpole) [14], Nepidae (water scorpions) [13, 14] were found to coexist with mosquito larvae in their aquatic habitats. Thus, malaria control interventions leveraging the interaction between mosquito larvae and aquatic predators might be established to reduce the survival of the larvae of malaria vectors, in particular *Anopheles funestus*, and eventually reduce malaria transmission [14].

Kweka et al. found evidence that natural predators in the aquatic habitats of *Anopheles gambiae *sensu lato (s.l.) could control populations and reduce malaria transmission in the highlands of western Kenya [16]. Other studies elsewhere have documented the efficiency of predators in controlling various mosquito species, such as *Aedes aegypti* [17] and *Culex quinquefasciatus* [18]. However, the studies did not address the effects of predators in areas where *An. funestus* is the predominant malaria vector, such as the Kilombero Valley, where it contributes to more than 85% of malaria transmission [19–21].

Thus, it remains unknown whether the same predation efficiency observed for other mosquito species would be observed for *An. funestus* [16–18]. Such information would be important in tackling the challenges associated with chemical-based interventions [11]. Therefore, this study aimed to evaluate the impact of three common predators recently identified in *An*. *funestus* aquatic habitats in rural southern Tanzania [14]. Specifically, this study assess the impacts of aquatic predators on (i) larval and adult *An. funestus* density and (ii) fitness traits of *An*. *funestus* mosquitoes (wing size, larval and adult survivals) in a semi-field system.

## Methods

### Study area

**Fig. 1:**
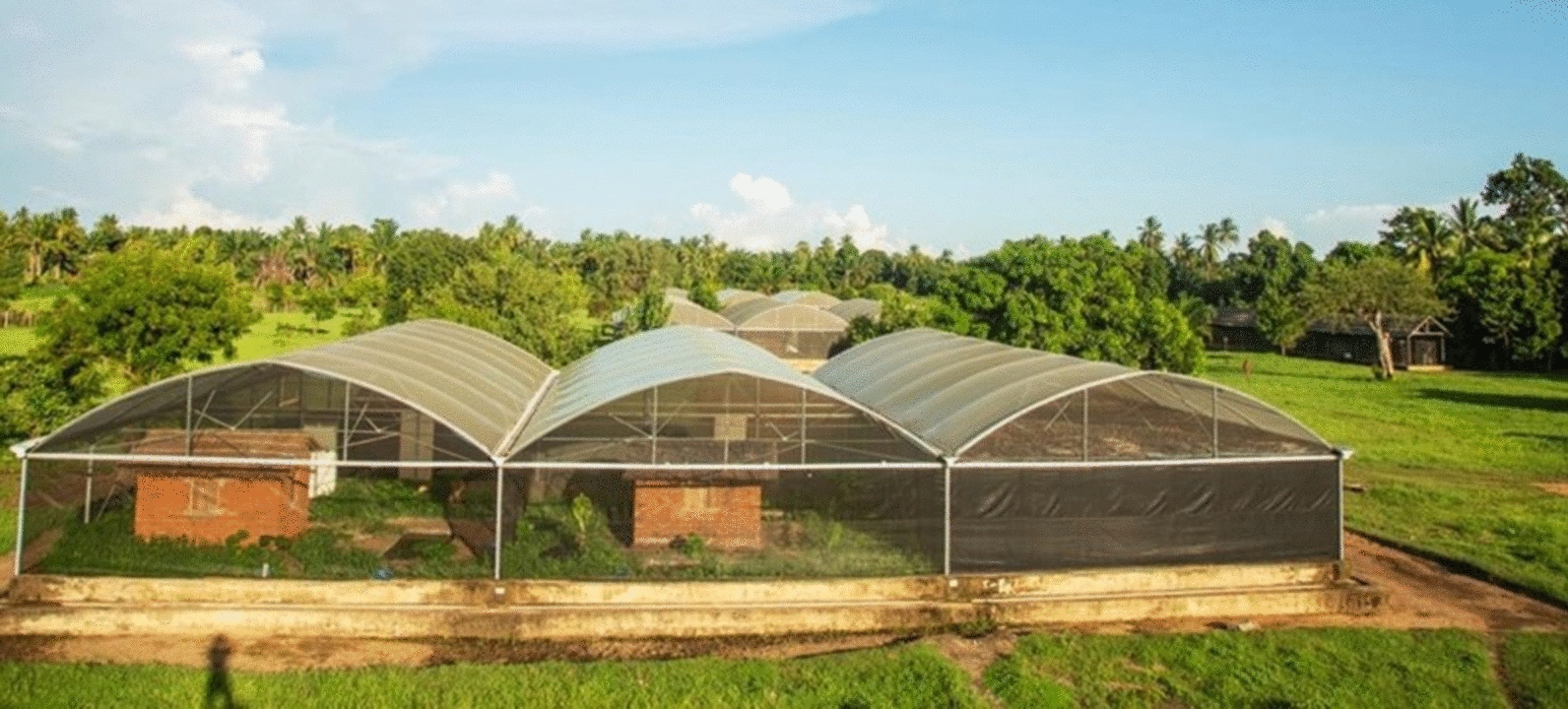
A large semi-field facility at Ifakara Health Institute “Mosquito City”

**Fig. 2:**
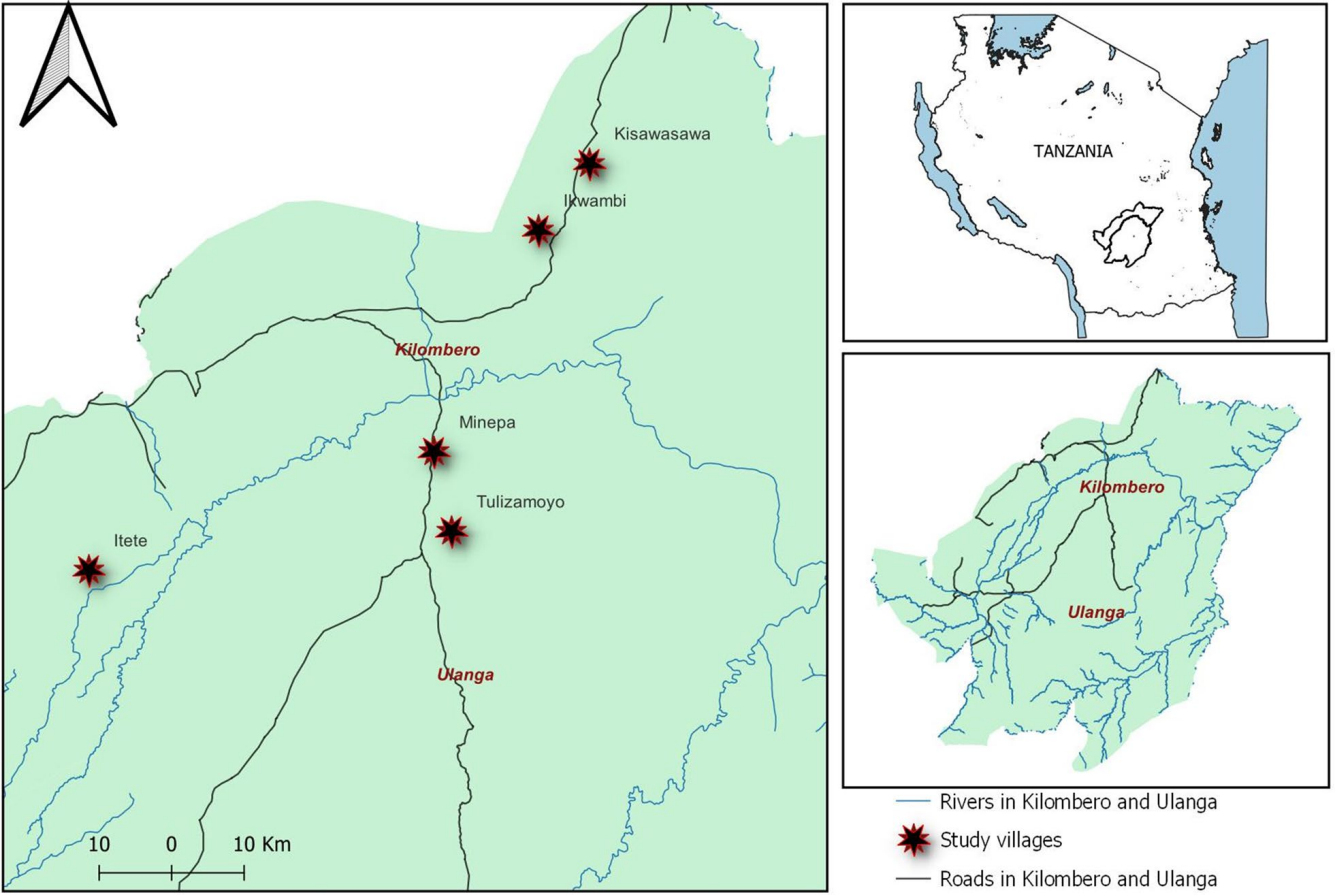
Map of villages where predators and *Anopheles funestus* were collected

The study was conducted at between June to November 2022, in a semi-field system facility known as ‘mosquito city’ [22], located at Kining’ina village in south eastern Tanzania. The semi-field system consists of three-chambered large screened-enclosures, measuring 28.8 m by 21 m, with walls made of UV-resistant shade netting, and a polyethylene roof mounted on a raised concrete platform [22]. This study used two chambers of this facility, each measuring 28.8 m by 7 m (Fig. 1). In addition, this large semi-field system has self-sustaining colonies of malaria vectors, experimental huts and vegetation to mimic natural environments. Aquatic predators and *An*. *funestus* larvae were collected from five villages in Kilombero and Ulanga districts Fig. 2; Itete (− 8.400°, 36.2506°), Tulizamoyo (− 8.35447°, 36.70546°), Minepa (− 8.2551°, 36.6839°), Ikwambi (− 7.97927°, 36.81630°), and Kisawasawa (− 7.89657°, 36.88058°). These villages were found to have both permanent and temporary water bodies including river stream, swamp, grounded pool. Water bodies from these villages were previously identified as harbouring common aquatic predators [14]. The area has received LLINs since the inception of distribution campaigns including the Tanzania National Voucher Scheme (TNVS) in 2004, mass distribution campaigns, Universal Coverage Campaigns (UCC) and School Net Programme (SNP) [23]. Previous studies in the area have mainly focused on characterizing *An. funestus* aquatic habitats [24], and identifying the common predators and factors influencing their abundance [14]. However, the interaction between *An. funestus* and these predators in their aquatic habitats is poorly understood.

### Predators and mosquito larvae collection

Predators were collected from natural *An. funestus* aquatic habitats using 10-L buckets and aquatic nets while *An. funestus* larvae were collected using 10-L buckets and pipettes as described by Mahenge et al. [14] and Nambunga et al. [24]. Both predators and *An. funestus* were transferred to buckets with water from their natural habitats and transported to the semi-field. In this experiment, only three predator families were collected, namely Aeshnidae (dragonflies), Coenagrionidae (damselflies), and Notonectidae (backswimmers). To avoid cannibalism between predators, each predator family was placed in a separate bucket. The *An. funestus* larvae were also placed in a separate bucket to prevent early predation. In the semi-field, *An. funestus* larvae were given fish food while predators were starved for 12-h as described by Kweka et al. [16], then were introduced into the artificial habitats.

### Preliminary evaluation within 24-h in a semi-field system to determine the appropriate larval instar stage for use in the study

A preliminary evaluation was performed in order to determine the larval instar stage that would be used in an experiment. Each predator family was assessed first for its preference and efficacy on all larval instars. This preliminary assessment identified the most suitable larval instar stage for the subsequent investigation. During the evaluation, all three-predator families were found to consume *An*. *funestus* larvae. In comparison to other larval instars, third larval instars were mostly consumed by all predators after 24-h of evaluation. Therefore, third instar larvae were used in all experiments in a semi-field.

### Experimental design in the semi-field and 24-h evaluation

Experiments were conducted in one habitat type which contained stone, 2 kg of sand and grass (Fig. 4a) which mimicked hiding structures found in natural habitats for *An. funestus* larvae against predators [16]. Five litres of water from the natural breeding habitats, 2 kg of soil, aquatic predators and the third instar of *An. funestus* larvae. The evaluation was taken after every 24-h in semi-field experiments.

**Fig. 3:**
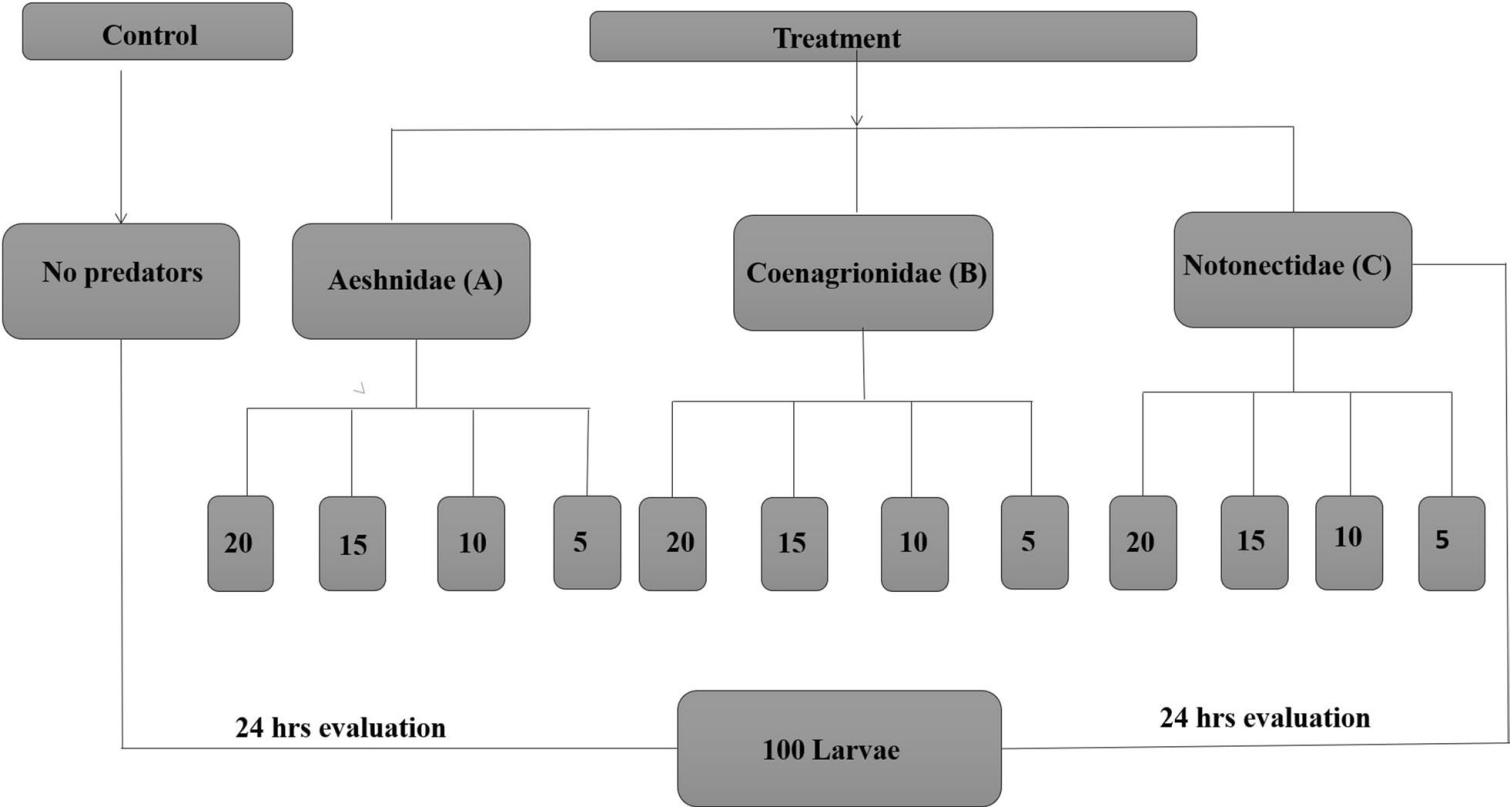
Experimental study design in the semi-field environment

In this experiment, three different predators were evaluated against a control (*An*. *funestus* larvae without predators). Four groups with different densities of predators were created to evaluate the effect of predatory density on different fitness traits of *An. funestus*. Different numbers of each predator type were placed in each group (i.e., 20, 15, 10 and 5). In each group a constant number of 100 *An. funestus* larvae were placed and another 100 *An. funestus* larvae were placed in the control arm. Each experimental group had three replicates. In all groups, the number of surviving larvae was counted and recorded every 24-h. This experiment is summarized in Fig. 3.

**Fig. 4:**
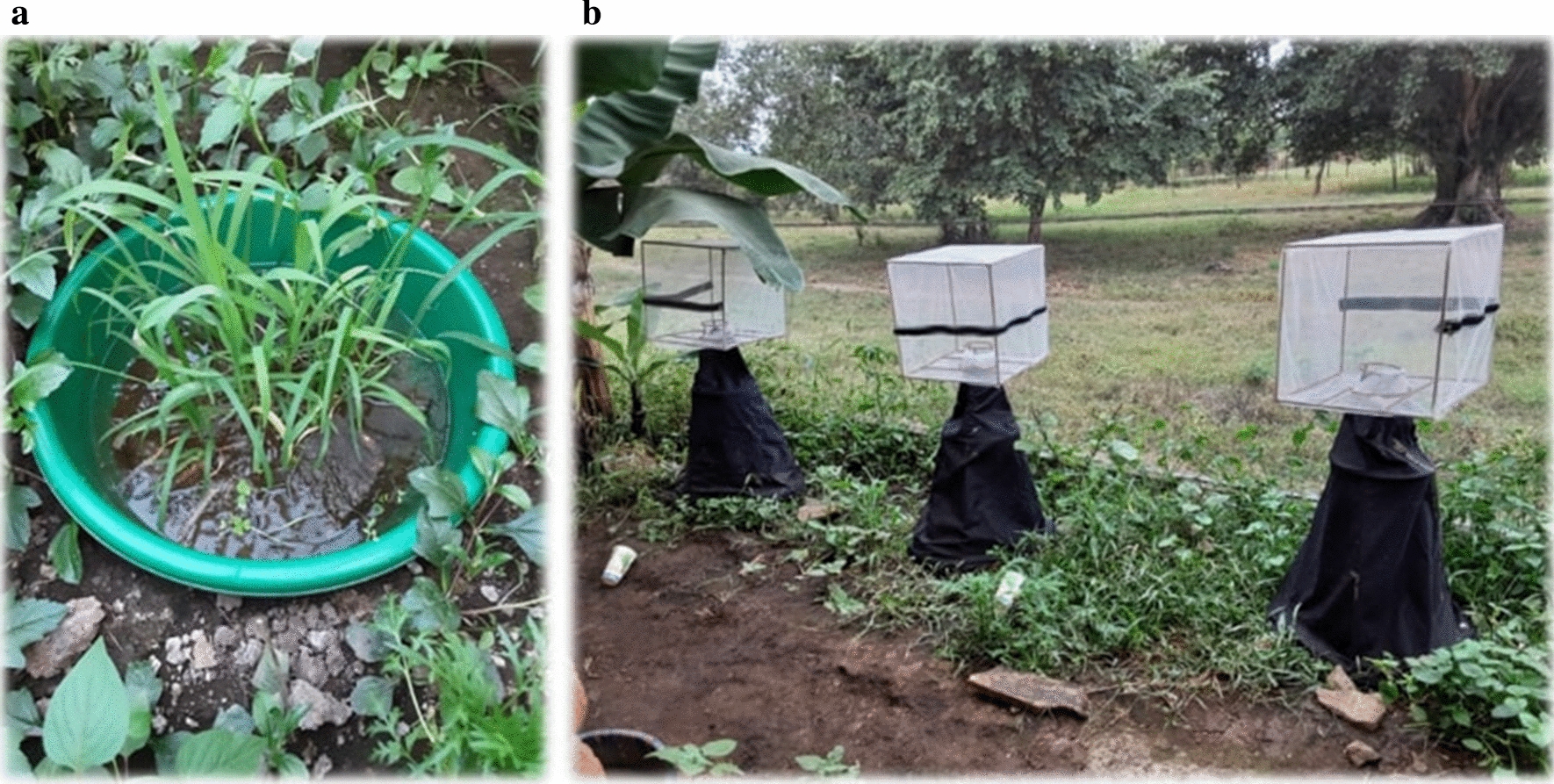
**a** Artificial habitats and **b** a trap on the top of artificial habitats for capturing the emerged mosquitos in a semi-field system

The remaining mosquito larvae were monitored until pupation, and then an emergence trap captured the emerging mosquitoes (Fig. 4b). The number of emerged mosquitoes in each group was recorded.

### Assessing the survival of adult mosquitoes

All mosquitoes emerged from both the treatment and control groups were transferred into 15 × 15 cm cages. They were then provided with 10% glucose solution-soaked cotton wool and their mortality was recorded every 24-h until all the mosquitoes had died.

### Assessing mosquito body size

Dead female mosquitoes were removed from the cages for wing measurement. A single wing of from each mosquito was placed on the microscope slide and a drop of distilled water was used for sticking it to the slide. Wing length was measured, using a micrometre ruler under a microscope, from the alula notch to the wing tip following procedures described by Lyimo et al*.* [25]. Wing length measurements were used as a proxy for mosquito body size.

### Statistical analysis

#### Assessing the impact of aquatic predators on *An. funestus* larval and adult density

Data were analysed using open source software R version 4.2.2 [26]. Generalized linear mixed model (GLMM) [27] with binomial variates was used to (i) estimate the Odds ratio and the absolute proportion of the number of larvae alive in each predator type, (ii) to assess the emergence rate of mosquitoes. The number of larvae alive and emerged mosquitoes in the artificial habitats was first assed in a group and later assessed individually. Results were presented as Odds Ratio (OR) with their corresponding 95% CI. Statistical significance was considered when P-value < 0.05. All plots were produced using *ggplot2* package [28]. The efficacy of each predator family was calculated as follows:

$$Efficacy=\frac{Control - Treatment}{Control}*100,$$

where “control” is the number of larvae alive in a habitat without predators, “treatment” is the number of larvae alive in habitats with predators.

#### Assessing the impact of aquatic predation on fitness traits of *An. funestus* mosquitoes (wing size and adult survivals)

Survival analysis was done using the Cox proportional hazard model using the *survival* package [29] to assess the odds of mortality for emerged mosquitoes (both males and females) for each predator type. The response variable was the observed time. Graphs were produced by *suveminer* package from a Kaplan Meir survival model [30]. Post-Hoc tests using the Tukey Honest Significance Difference (THSD) was used to assess in the mean differences in wing size of mosquitoes emerged from different predators and the control.

## Results

### Impact of predators on larvae and adult density

**Fig. 5:**
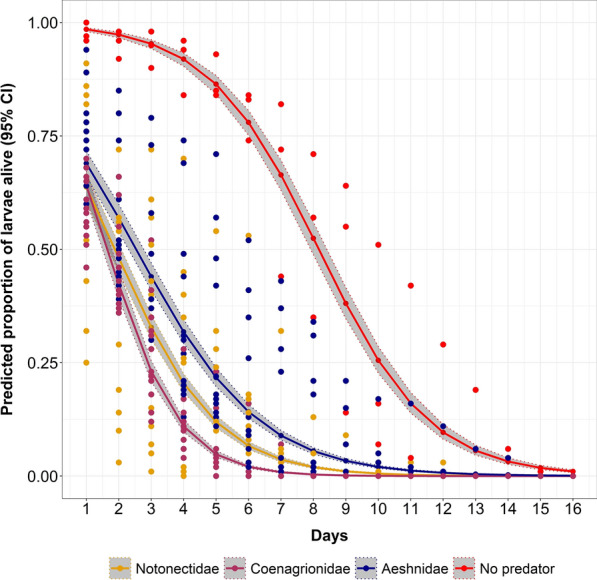
Predicted proportion of *Anopheles funestus* larvae alive as estimated by generalized linear mixed effect model. The coloured lines represent different predators used, dots are observed values, shaded areas are the 95% confidence intervals

**Fig. 6:**
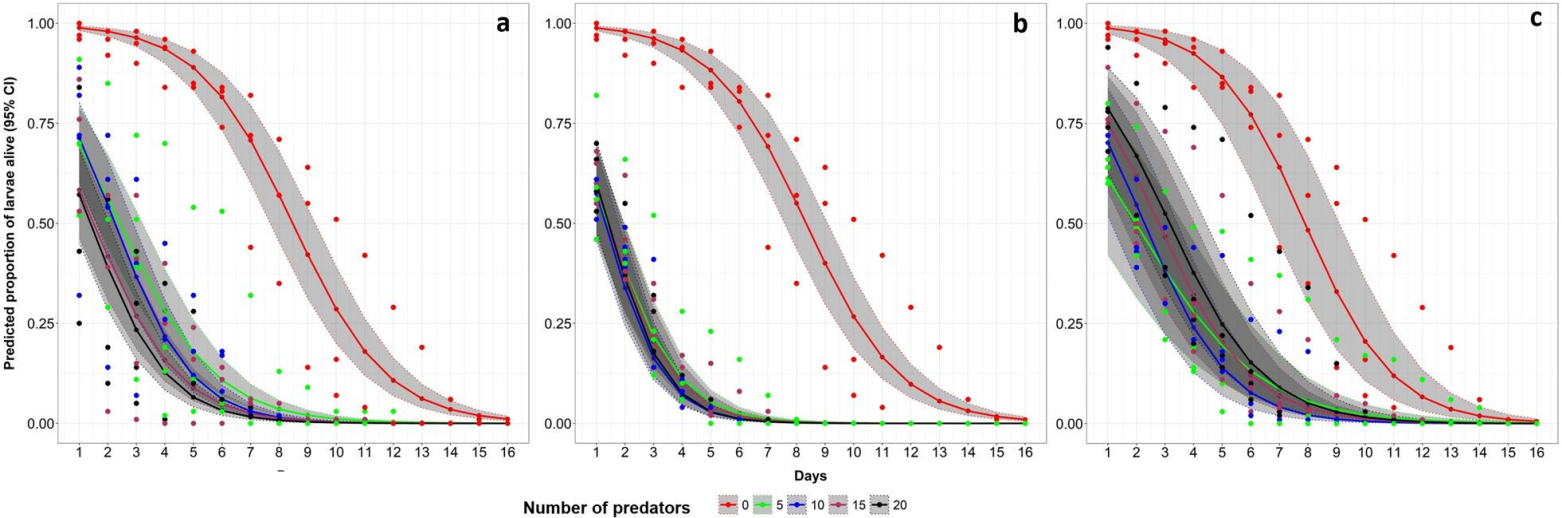
Predicted proportion of larvae alive in different predator families **a** Notonectidae (backswimmers), **b** Coenagrionidae (damselflies) and **c** Aeshnidae (dragonflies) predators. The coloured lines represent different numbers of predators used, dots are the observed values and shaded areas are the 95% confidence interval

The predation impact of the three predator families varied significantly in both treatment and control (P < 0.001, Table 1, Fig. 5). The proportion of larvae alive was high in habitats with a small number of Notonectidae (backswimmers) and Coenagrionidae (damselflies) (P < 0.001, Table 2, Fig. 6). Contrarily, Aeshnidae (dragonflies), larval survival was high in treatments with a high number of predators and low in habitats with a low number of predators (P < 0.001, Table 2, Fig. 6).

**Table 1:** The efficacy of different predators in reducing the survival of *An. funestus* larvae in the semi-field experiment settings

Predator	Absolute proportion	OR [95% CI]	Relative reduction (%)	P-value
Control	47.85	1		
Notonectidae (backswimmers)	11.96	0.15 [0.14, 0.16]	75.01	< 0.001
Coenagrionidae (damselflies)	9.29	0.11 [0.10, 0.12]	80.59	< 0.001
Aeshnidae (dragonflies)	16.26	0.21 [0.20, 0.23]	66.02	< 0.001

Absolute proportions as estimated from generalized linear mixed effect model

**Table 2:** Odds ratio, absolute proportion and their corresponding 95% confidence intervals showing the proportion of larvae alive when exposed to different number of predators

Predator	Number of predators	Absolute proportion [95% CI]^a^	OR [95% CI]	P-value
Notonectidae (backswimmers)	0	50.66 [43.75, 57.53]	1	
	5	14.80 [11.61, 18.67]	0.17 [0.15, 0.19]	< 0.001
	10	12.86 [10.03, 16.35]	0.14 [0.13, 0.16]	< 0.001
	15	10.06 [7.78, 12.92]	0.11 [0.10, 0.12]	< 0.001
	20	9.03 [6.96, 11.65]	0.10 [0.09, 0.11]	< 0.001
Coenagrionidae (damselflies)	0	48.87 [44.25, 53.5]	1	
	5	9.22 [7.70, 11.0]	0.11 [0.09, 0.12]	< 0.001
	10	7.86 [ 6.53, 9.43]	0.09 [0.07, 010]	< 0.001
	15	8.91 [7.44, 10.63]	0.10 [0.09, 0.11]	< 0.001
	20	8.29 [6.90, 9.94]	0.09 [0.08, 0.11]	< 0.001
Aeshnidae (dragonflies)	0	45.18 [35.20, 55.55]	1	
	5	14.57 [10.10, 20.58]	0.21 [0.19, 0.23]	< 0.001
	10	13.22 [9.11, 18.80]	0.18 [0.17, 0.20]	< 0.001
	15	16.04 [11.18, 22.48]	0.23 [0.21, 0.25]	< 0.001
	20	18.04 [12.67, 25.04]	0.27 [0.24, 0.29]	< 0.001

^a^Absolute proportions as estimated from generalized linear mixed effect mode

### Impact of predators on the adult’s density

**Fig. 7:**
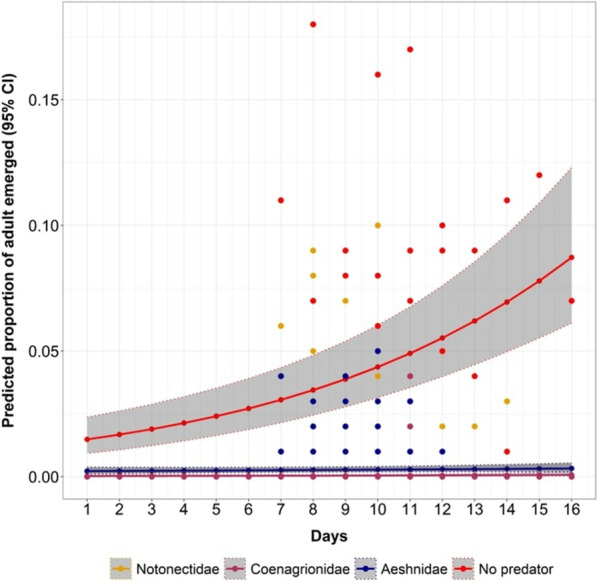
Predicted proportion of mosquito larvae emerged in all the different predator families. Dots are observed values; shaded areas are the 95% confidence interval

There were significant differences in the emerged mosquitoes from the treatment group and the control group (P < 0.001, Table 3, Fig. 7). Moreover, the proportion of mosquitoes that emerged from the habitat containing Coenagrionidae (damselflies) was lower compared to those that emerged from the other predators (P < 0.001, Table 3, Fig. 7).

**Table 3:** The efficacy of different predators in reducing the emergence of *An. funestus* adult*s* in semi-field experiment settings

Predator	Absolute proportion	OR [95% CI]	P-value
Control	4.20	1	
Notonectidae (backswimmers)	0.27	0.06 [0.05, 0.08]	< 0.001
Coenagrionidae (damselflies)	0.04	0.01 [0.0, 0.02]	< 0.001
Aeshnidae (dragonflies)	0.27	0.06 [0.05, 0.08]	< 0.001

### Assessing the impact of predators on the fitness parameters of the mosquitoes (wing size, survival and fecundity)

#### Survival time of emerged mosquitoes

**Fig. 8:**
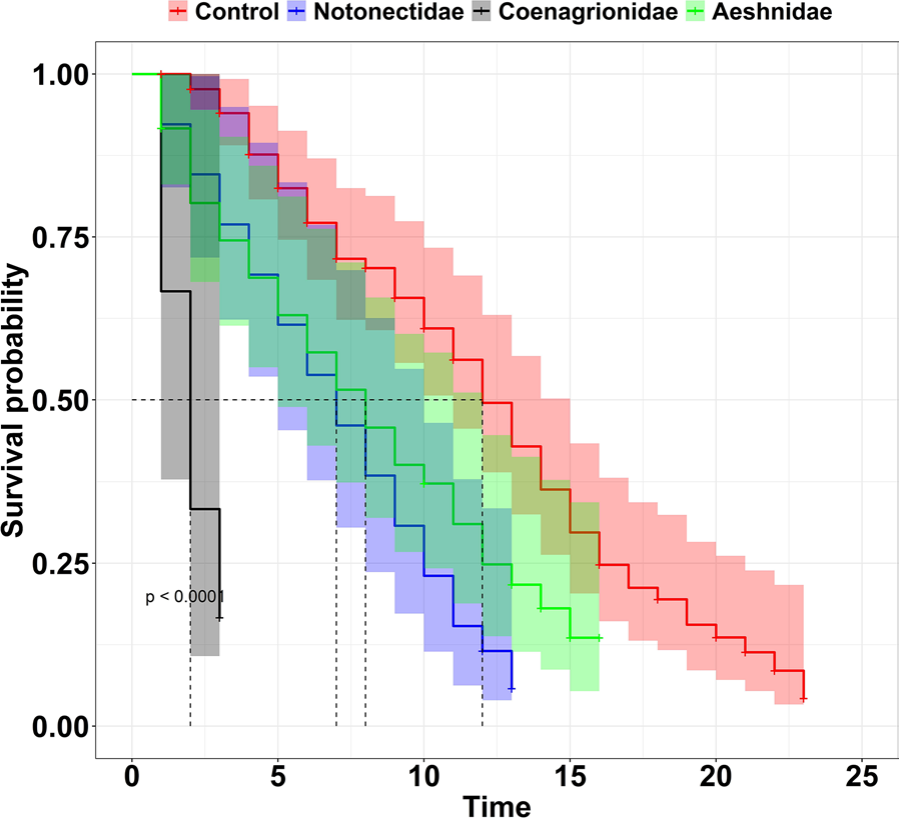
Survival of *Anopheles funestus* mosquitoes from the different predator families. Full lines represent the survival function as estimated from fitting the Kaplan Meir survival model and shaded area with different color express the 95% CI. Dotted grey horizontal and vertical lines show the median survival days of mosquitoes at 50% probability

**Fig. 9:**
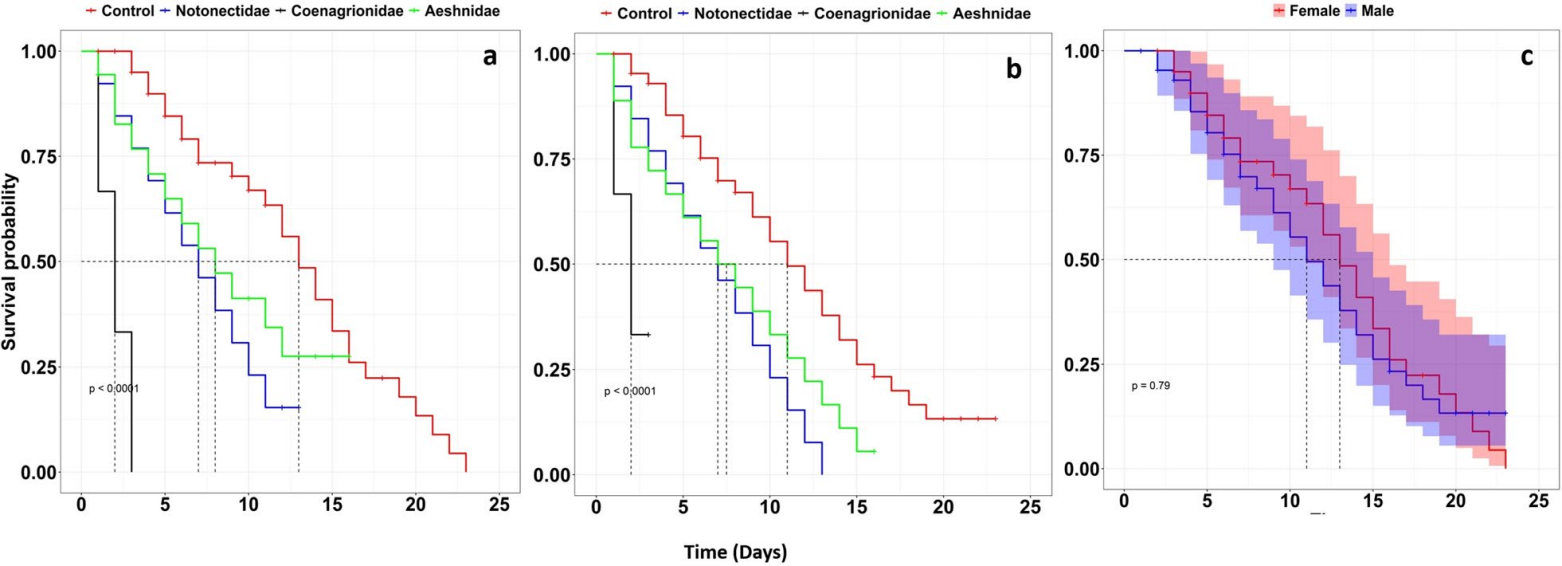
Survival of **a** female *Anopheles funestus*, **b** male *Anopheles funestus* and **c** female and male mosquitoes emerged on control group. Full lines represent the survival function as estimated from fitting the Kaplan Meir survival model and shaded area express 95% CI. Dotted grey horizontal and vertical lines show the median survival days of mosquitoes at 50% survival probability

**Fig. 10:**
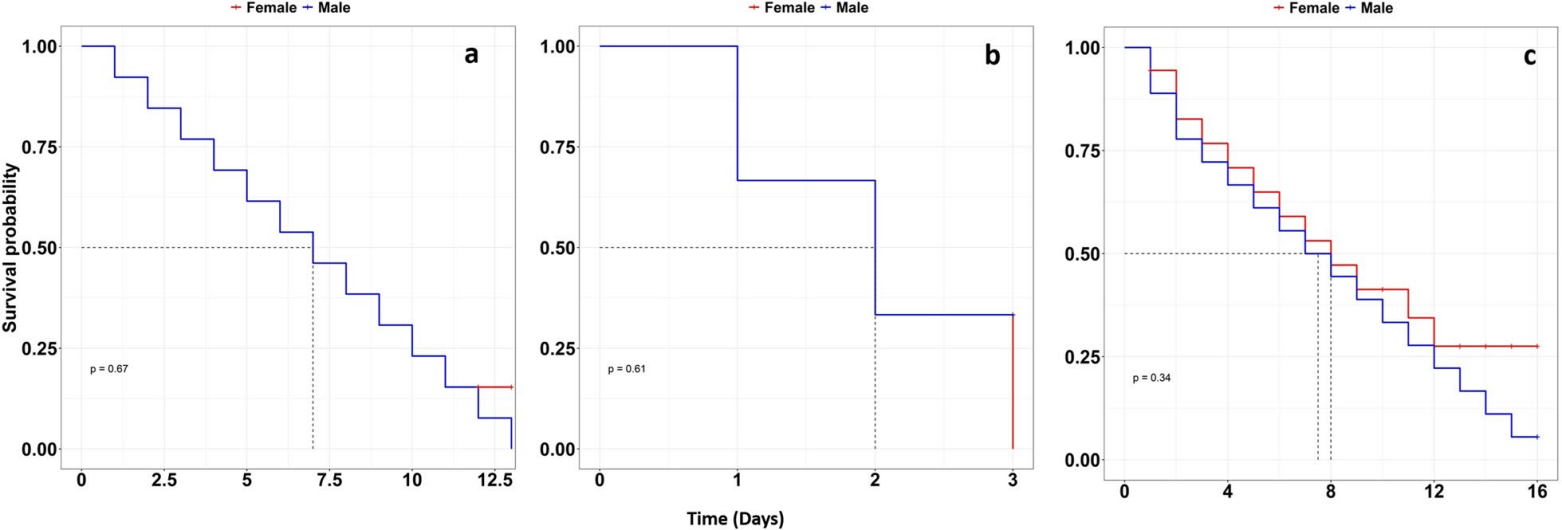
Survival of male and female *Anopheles funestus* adults that emerged from **a** Notonectidae (backswimmers), **b** Coenagrionidae (damselflies) and **c** Aeshnidae (dragonflies). Full lines represent the survival function as estimated from fitting the Kaplan Meir survival model and shaded area express 95% CI. Dotted grey horizontal and vertical lines show the median survival days of mosquitoes at 50% survival probability

Cox regression analysis showed a significant difference between the median survival of mosquitoes that emerged from the control group and those that emerged from all treatment groups (i.e., their larvae were exposed to Coenagrionidae (damselflies), Notonectidae (backswimmers) and Aeshnidae (dragonflies). The analysis showed that *An*. *funestus* mosquitoes emerged from Coenagrionidae (damselflies) were seventeen times more likely to die earlier [HR = 17.31 (95% CI 6.24, 48.02), P < 0.001] compared to the control group. Those exposed to Notonectidae (backswimmers) and Aeshnidae (dragonflies) were 2.82 [HR = 2.82 (1.70, 4.66), P < 0.001] and 1.88 [HR = 1.88 (1.19, 2.97), P < 0.001] times more likely to die compared to the control group respectively (Table 4, Fig. 8). In addition, there was no significant difference between the survival of male and female mosquitoes emerged from different predator types (P > 0.05, Table 5, Figs. 9 and 10). Overall, mosquitoes in the control group took a median of 12 (95% CI 11–15) days to die while those exposed to predators took less than 10 days (P-value < 0.001, Table 4).

**Table 4:** Hazard ratios (HR) and median survival days of adult *Anopheles funestus* emerged from different predator types and their corresponding 95% CI and P-value

Predator	Median [IQR]	HR [95% CI]	P-value
Control	12 [11, 15]	1	
Notonectidae (backswimmers)	7 [5, 10]	2.82 [1.70, 4.66]	< 0.001
Coenagrionidae (damselflies)	2 [1, 2]	17.31 [6.24, 48.02]	< 0.001
Aeshinidae (dragonflies)	8 [5, 12]	1.88 [1.19, 2.97]	< 0.001

**Table 5:** Hazard ratios (HR) and median survival days of adult male and female *Anopheles funestus* emerged from different predator types and their corresponding 95% CI and P-values

Predator	Sex	Median [IQR]	HR [95% CI]	P-value
Notonectidae (backswimmers)	Females	7 [4, 10]	1	0.683
	Males	7 [4, 11]	1.18 [0.53, 2.64]	
Coenagrionidae (damselflies)	Females	2 [1, 2]	1	0.657
	Males	2 [1, 2]	0.67 [0.11, 3.99]	
Aeshnidae (dragonflies)	Females	8 [5, 13]	1	0.353
	Males	7.5 [4, 13]	1.42 [0.68, 2.97]

#### Mosquito body size

**Fig. 11:**
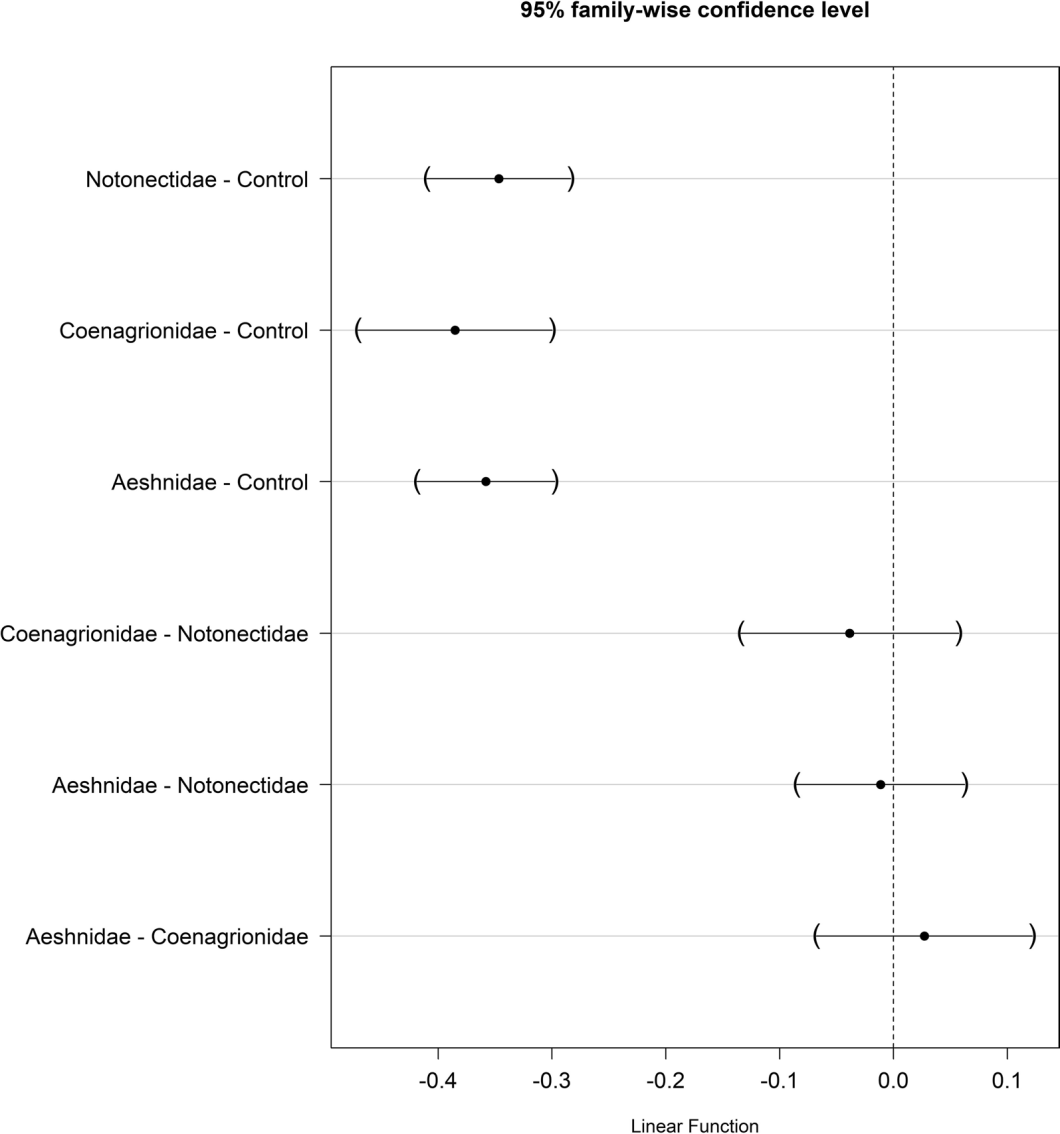
A Tukey’s post hoc test showing pair-wise comparison of the mean wing sizes of *Anopheles funestus* adults mosquitoes emerged from different predator type and the control group

**Fig. 12:**
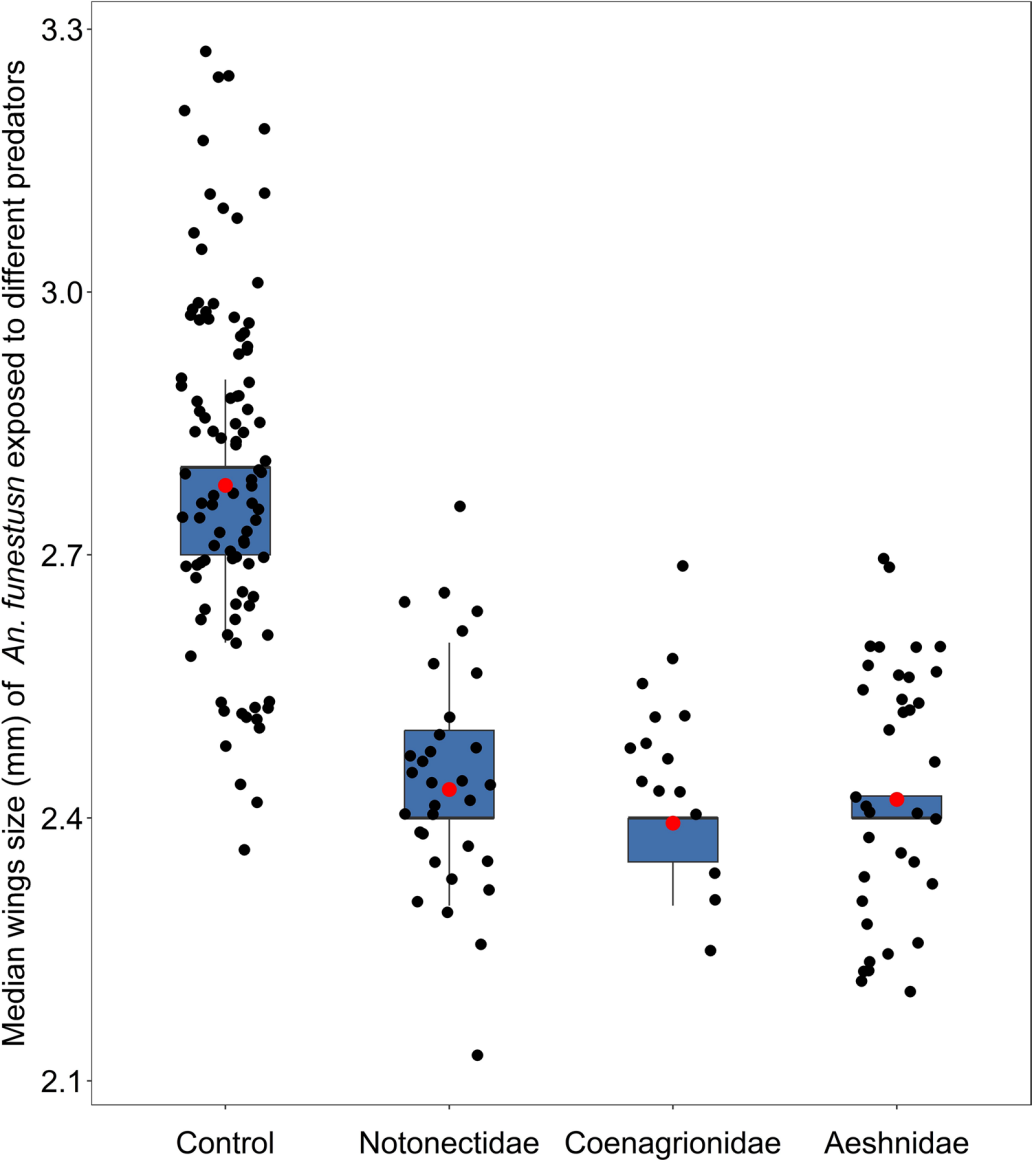
Median wing size of adult female *Anopheles funestus* emerged from different predator type

A Tukey’s post hoc test showed that the *An. funestus* mean wings size varied significantly between the groups i.e., treatment (with different predators’ families) and the control (P < 0.05, Table 6, Figs. 11 and 12), while there was no difference in wing size in mosquitoes emerged from Notonectidae (backswimmers), Coenagrionidae (damselflies) and Aeshnidae (dragonflies) treatments (P > 0.05, Figs. 11 and 12).

**Table 6:** Mean wing sizes of females *Anopheles funestus* after being exposed to different predators during larvae stages

Predator type	Predicted mean [2se]^a^
Control	2.779 [0.0127]
Notonectidae (backswimmers)	2.433 [0.0214]
Coenagrionidae (damselflies)	2.394 [0.0313]
Aeshinidae (dragonflies)	2.421 [0.0202]

^a^Mean values estimated from generalized linear mixed models

## Discussion

This is the first study which assesses the impact of predators on larval and adult density as well as on the fitness characteristics of *An. funestus*. Coenagrionidae (damselflies) and Notonectidae (backswimmers) were more efficient predators of *An*. *funestus* than Aeshnidae (dragonflies). Similarly, studies reported the efficiency of Notonectidae (backswimmers) on other mosquito species [16, 31]. Earlier study reported the direct impact of aquatic predators on *An. gambiae* larvae and the number of eggs laid by adult *An*. *gambiae* mosquitoes [32].

Using an epidemiological model, Roux et al. [33] demonstrated that the trait alteration by larval predation stress can decrease malaria transmission. This is primarily attributed to lower fecundity and longevity, which are important factors of the mosquitoes’ vectorial capacity. Additionally, other studies have suggested that the direct and indirect effects of predators on vector life-history traits and consequently on mosquito vectorial capacity and consequently, the transmission of pathogens [34, 35].

In recent years, Aeshnidae (dragonflies) have been shown to be efficient in reducing *An*. *arabiensis* [36] and *Aedes aegypti* [37]. The current study tested their efficacy on *An*. *funestus* larvae and found them to be the least efficient when compared to other predators tested Coenagrionidae (damselflies) and Notonectidae (backswimmers). This findings corroborate with Kweka et al., on *An*. *gambiae*, which demonstrated that Aeshnidae (dragonflies) were inefficient when compared to Notonectidae (backswimmers) and other predators evaluated together [16]. This could be due to differences in eating preferences and hunting modes among predator species, though it is difficult to determine this, because the current study did not morphologically identify these predators into species level.

During the preliminary assessment, it was observed that all predators significantly consumed *An*. *funestus* larvae of intermediate size (instar 3), while showing less interest in small instars (Instar 1 and Instar 2) and larger larvae (Instar 4). These findings align with previous studies conducted on various mosquito species in different settings [16, 35, 38, 39]. The selection of larvae instar 3 could potentially be attributed to the challenges predators face in handling the other instar sizes particularly very small and larger ones. In the semi-field experiment, daily and overall predation rates (consumption, pupation rate, emergence and adult survival rate) varied depending on predator families and densities. The rates of consumption varied among predators during the 24-h of evaluation. Consumption was higher in habitats with a higher number of predators of Coenagrionidae (damselflies) and Notonectidae (backswimmers). On the other hand, Aeshnidae (dragonflies) consumption rate was highest in habitats with a small number of these predators. This could be due to higher inter-species competition in habitats with a higher number of Aeshnidae (dragonflies) than in habitats with a lower number. Therefore, this may increase the rate of consumption in lower numbers of Aeshnidae (dragonflies). Among the evaluated predators, Coenagrionidae (damselflies) had the greatest impact on *An*. *funestus* larvae in terms of consumption rate, emergence, adult mosquito survival, and wing size. The number of mosquitoes that emerged was lower in Coenagrionidae (damselflies) than in Notonectidae (backswimmers) and Aeshnidae (dragonflies) treatments. In general, there is a significant difference in the number of mosquitoes emerging in predators versus control.

Overall, all mosquito larvae exposed to aquatic predators had a lower adult survival rate, but among all emerged mosquitoes, only that emerged from Coenagrionidae (damselflies) died earlier than those that emerged from Notonectidae (backswimmers) and Aeshnidae (dragonflies). This could be because each predator has a different mode of action on mosquito larvae and different feeding behaviour. Nevertheless, when comparing the effects of all three predators on survival of adult male and female mosquitoes there was no significant difference. This indicates that, predators have an equal likelihood of affecting both males and females *An*. *funestus*, hence positive impact on malaria vector reduction.

One advantage of using biological control methods is that they can target mosquito species at low densities, with no impact on non-targeted organisms. Also, it is easy to implement in the field [40]. However, before introducing or implementing these predators as a bio-control of *An*. *funestus* mosquito larvae, it is important to understand their ecological implications because these could result in other negative impacts on the ecosystem if it is not well assessed.

This study effectively assessed the impact of aquatic predators on the *An. funestus* larval density and the impact of predation on the fitness parameters (body size and adult survival). However, the current study fails to assess the predation impact on fecundity. Therefore, rigorous and robust experiments should be done to assess the impacts of predators on the fitness parameters of adult mosquitoes including the fecundity. Another potential limitation of this study is the lack of assessment of the interaction of multiple predators on *An. funestus* larvae. In the natural aquatic environments, it is common for multiple predator species to coexist, and their combined effect on prey populations can differ from the individual effects of each predator. This study did not consider the potential interactions between different predator species; thus, this study may have overlooked important factors that could influence the population dynamics of *An. funestus* larvae. Also, in the semi-field system, the 5 L artificial habitat which is different from the large habitat, sand, grass and stones were added to mimic the natural aquatic habitat of *An. funestus* and predators. It is recognized that larger aquatic habitats might offer *An*. *funestus* larvae greater opportunities to avoid predation due to increased space and potentially diverse habitat structures.

The current study focused solely on third instar larvae and small aquatic habitats, this might not represent the full range of the larval stages of *An. funestus* and their natural habitats. Since the predation dynamics can vary with different instars, and size of the habitats larvae’s ability to escape or hide from predators. Future research may investigate a broader range of larval instars and consider using larger water bodies to better represent natural conditions. This would provide a more comprehensive understanding of predator–prey relationships across different life stages in a natural environment. Additionally, the study did not assess the impacts of predators throughout different seasons. Environmental conditions, such as temperature, precipitation, and resource availability, can vary significantly throughout the year, affecting both predator and prey populations. Predators may exhibit different behaviour and prey preferences in different seasons, which could impact the population dynamics of *An. funestus* larvae. It is important for further studies to consider these seasonal variations so as to draw comprehensive conclusions about the long-term effects of predators on *An. funestus* larvae.

## Conclusion

This study shows that overall predation impact of the evaluated predators was high, and all three predators were able to reduce the larval and adult density of *An. funestus* mosquitoes. Additionally, these predators significantly reduced the emergence rate and affected the fitness traits of emerged *An. funestus* mosquitoes, including the survival and wings size. Furthermore, the findings of this study provide valuable insights relevant for understanding the effectiveness of these predators against *An*. *funestus* larvae and can be used as baseline for future studies in the natural aquatic environments in rural-Tanzania and other regions with comparable eco-epidemiological conditions.

## Data Availability

All data for this study will be available upon request.
